# Comparison of Post-Cesarean Pain Perception of General Versus Regional Anesthesia, a Single-Center Study

**DOI:** 10.3390/medicina59010044

**Published:** 2022-12-27

**Authors:** Danka Mostic Stanisic, Nevena Kalezic, Aleksandar Rakic, Nina Rajovic, Tatjana Ilic Mostic, Jelena Cumic, Jelena Stulic, Ivana Rudic Biljic Erski, Nevena Divac, Natasa Milic, Radan Stojanovic

**Affiliations:** 1 Clinic for Gynecology and Obstetrics, Faculty of Medicine, University of Belgrade, 11000 Belgrade, Serbia; 2 Clinic for Gynecology and Obstetrics, Clinical Center “Dragisa Misovic–Dedinje”, 11000 Belgrade, Serbia; 3 Department of Anesthesiology, Faculty of Medicine University of Belgrade, 11000 Belgrade, Serbia; 4 Clinic of Gynecology and Obstetrics “Narodni Front”, 11000 Belgrade, Serbia; 5 Institute for Medical Statistics and Informatics, Faculty of Medicine University of Belgrade, 11000 Belgrade, Serbia; 6 Department of Anesthesiology, Clinic of Gynecology and Obstetrics, Clinical Centre of Serbia, 11000 Belgrade, Serbia; 7 Institute for Pharmacology, Clinical Pharmacology and Toxicology, Faculty of Medicine, University of Belgrade, 11000 Belgrade, Serbia; 8 Department of Internal Medicine, Division of Nephrology and Hypertension, Mayo Clinic, Rochester, 55904 MN, USA

**Keywords:** general anesthesia, regional anesthesia, cesarean section, post-cesarean pain, SF-MPQ, VAS, pain attributes questionnaire, lactation, oral intake, mobilization

## Abstract

*Background and Objectives*: Pain during and after the procedure remains the leading concern among women undergoing cesarean section. Numerous studies have concluded that the type of anesthesia used during a cesarean section undoubtedly affects the intensity and experience of pain after the operation. *Materials and Methods*: This prospective cohort study was conducted at the Clinic for Gynecology and Obstetrics, Clinical Center “Dragisa Misovic—Dedinje”, Belgrade, Serbia. Patients at term pregnancy (37–42 weeks of gestation) with an ASA I score who delivered under general (GEA) or regional anesthesia (RA) by cesarean section were included in the study. Following the procedure, we assessed pain using the Serbian McGill questionnaire (SF–MPQ), Visual Analogue Scale (VAS) and the pain attributes questionnaire at pre-established time intervals of 2, 12, and 24 h after the procedure. Additionally, time to patient’s functional recovery was noted. We also recorded the time to the first independent mobilization, first oral intake, and lactation establishment. *Results*: GEA was performed for 284 deliveries while RA was performed for 249. GEA had significantly higher postoperative sensory and affective pain levels within intervals of 2, 12, and 24 h after cesarean section. GEA had significantly higher postoperative VAS pain levels. On pain attribute scale intensity, GEA had significantly higher postoperative pain levels within all intervals. Patients who received RA had a shorter time to first oral food intake, first independent mobilization, and faster lactation onset in contrast to GEA. *Conclusions*: The application of RA presented superior postoperative pain relief, resulting in earlier mobilization, shorter time to first oral food intake, and faster lactation onset in contrast to GEA.

## 1. Introduction

Despite the effort and recommendations, almost one in five children are born in an operating room [1]. Pain during and after the procedure remains the leading concern among women undergoing cesarean section [2,3]. This fear appears to be universal, regardless of age, race, education level, or obstetric factors (i.e., parity or previous delivery by cesarean section) [4]. Post-cesarean pain is a complex personalized experience. It is a subjective feeling that is described by using different characteristics (quality, localization, intensity, emotional impact) and can be defined in two ways: verbal description (descriptor with more words) and/or numerical display [5].

Acute pain after a cesarean section leads to a delayed recovery of patients, as well as a longer hospital stay [6]. In addition, acute pain after a cesarean section is associated with a higher risk of developing chronic pain [7,8]. Higher pain scores increase the risk of postpartum depression [9]. It is also known that pain after a cesarean section affects the timing and quality of bonding between mother and child [10]. All these facts unquestionably indicate that the pain after a cesarean section can have extensive psychological and socio-economic consequences. Nikolajsen et al. showed that 6% of women complained of pain interfering with their quality of life, while 12% of women were still feeling pain after 10 months of giving birth [11].

Numerous studies have concluded that the type of anesthesia during a cesarean section undoubtedly affects the intensity and experience of pain after the operation [12]. Anesthetic, maternal, and fetal factors, together with the anesthesiologist’s judgment and patient’s preference determine which type of anesthesia will be used for cesarean section [13]. It is difficult to predict postoperative pain severity or the patient’s reaction to the regimen. An optimal post-cesarean analgesic regimen should be cost-effective and applicable, meaning that it should be adjusted to a patient’s preferences (with minimal effect on personnel workload), result in a low incidence of side-effects and complications, should not interfere with the maternal care of the newborn, and ensure a minimal amount of drugs passing into breast milk [14]. We hypothesized that regional anesthesia (RA) may be superior in postoperative pain relief, thus resulting in earlier mobilization, shorter time to first oral food intake, and faster lactation onset in contrast to cesarean section performed under general anesthesia (GEA). Therefore, in this study, we aimed to explore the differences in postoperative pain among women delivering by cesarean section performed under GEA and RA. Additionally, time to functional recovery, including time to lactation establishment, oral intake, and early mobilization, was assessed.

## 2. Materials and Methods

### 2.1. Study Population

This prospective cohort study was conducted at the Clinic for Gynecology and Obstetrics, Clinical Center “Dragisa Misovic-Dedinje”, Belgrade, Serbia. Institutional Review Board (IRB) of Medical Faculty, University of Belgrade (29/IX-9) and IRB of Clinical Center “Dragisa Misovic-Dedinje” (01-8127/16) approved the study. Every patient gave informed consent before the surgery. Patients at term pregnancy (37–42 weeks of gestation), with ASA II score (American Society of Anesthesiologists’ score—a subjective assessment of a patient’s overall health ranging from I to V), who delivered by cesarean section, were included in the study. Type of anesthesia used for cesarean section was determined either based on anesthesiologist’s judgment or patient’s preference. Both scheduled and emergent cesarean sections were included in the study. If patients did not receive standard anesthetic protocol, had some form of complicated pregnancy, were transferred to the intensive care unit, were diagnosed with chorioamnionitis (the latter because their pain scores may have been affected by a concurrent infection or could intensify the post-surgical pain), or did not provide the written consent, they were excluded.

### 2.2. Anesthetics Protocols

The anesthesiologist monitored non-invasive blood pressure, electrocardiogram, and pulse oximetry for all patients during the procedure. Before the induction of anesthesia, every patient received Ringer lactate solution (500 mL).

GEA protocol: After the adequate preoperative preparation for the cesarean section, and before the induction in GEA, patients were adequately positioned to avoid aortocaval compression and its effect on hemodynamics. We placed a high-flow peripheral line and, in case of potential massive bleeding, several venous lines. Non-invasive basic monitoring included blood pressure measurement, ECG monitoring, pulse oximetry, and capnography. We performed preoxygenation to increase the oxygen reserve in the lungs during apnea (inhalation of 100% oxygen for two minutes, which provides more than 95% complete preoxygenation in pregnant women). After preoxygenation, we started administering the induction drug propofol in a dose of 2.3 mg/kg intravenously. As a muscle relaxant for induction, we used succinylcholine (a depolarizing muscle relaxant for rapid induction in a dose of 1.5 mg/kg). Laryngoscopy was performed using direct laryngoscopes or video laryngoscopes. Smaller diameter tubes with inner diameter of 6–7 mm were placed. After induction and intubation, anesthesia was maintained with a mixture of inhalational anesthetic gases and oxygen. Nitric oxide, which serves to maintain GEA, was made up 50% of the pre-extraction mixture and 67% of mixture after the baby was extracted. In cases of emergencies and fetal endangerment, 100% oxygen mixed with sevoflurane was applied until the moment of extraction. Sevoflurane was applied in dose of 0.6 MAC-A so that it would have no effect on the relaxation of the uterus and the occurrence of atony. Fentanyl 3 µ/kg and rocuronium 0.5 mg/kg were applied after baby extraction and umbilical cord clamping for placenta extraction. After surgery, the neuromuscular block reversal was performed with a mixture of prostigmine and atropine. We did not use sugammadex in any patient. After careful planning and preparation, extubation was performed in a fully awake patient who responded to voice commands, maintained adequate blood oxygen saturation, and had satisfactory respiratory volume and protective reflexes. Postoperative monitoring was mandatory.

Spinal anesthesia (SA) protocol: The spinal anesthetic (SA) was induced by hyperbaric bupivacaine 12 mg and fentanyl 0.01 mg. After the spinal injection, blood pressure was measured every minute for the first 10 min and then every 3 min until the end of the procedure. By protocol, any reduction in systolic pressure by at least 10% from preoperative pressure or below 100 mmHg would be treated with intravenous ephedrine (3–6 mg).

Epidural anesthesia (EA) protocol: After the insertion of the epidural catheter and the application of the test dose, isobaric bupivacaine 0.5% (0.5 mg per 10 cm height) and fentanyl 0.05 mg were injected. Removal of the epidural catheter was performed 24 h after its placement. Epidural catheter was used only in patients where cesarean section was performed under EA and a combined SA-EA approach was not used.

In the recovery room, the patients stayed for an hour after the cesarean section. A non-invasive blood pressure, heart rate, and pulse oximetry measurement was taken every 15 min for the first hour, and every 30 min for the second hour, within the postoperative observation area. Details regarding non-invasive measurements have been described in detail elsewhere [15].

Postoperative pain therapy was managed by multimodal approach using intravenous analgesics [16]. All patients received 1 mg/kg of diclofenac every 8 h for 24 h after surgery. In addition, intravenous diclofenac was available upon request without a time limit if patients reported inadequate analgesia via epidural catheter; however, there was a restriction on the total dose, as recommended by the manufacturer. GEA patients received Tramadol 100 mg and, in addition, Acetaminophen 1 gr optionally every 8 h for 24 h after surgery. Tramadol was received by 98% of GEA patients (280/284), and Acetaminophen was received by 78.5% of GEA patients (223/284), in contrast to no RA patients who received Tramadol/Acetaminophen (0/249).

### 2.3. Pain Assessment

After the surgery, an independent anesthesiologist interviewed each patient at pre-established time intervals of 2, 12, and 24 h. Analgesics were always prescribed only after patients had assessed their pain. Following the study design, we assessed pain using the shortened version of the Serbian McGill questionnaire (SF–MPQ) [5]. To complete the assessment, we also used the Visual Analogue Scale (VAS) and the pain attributes questionnaire. The SF-MPQ consists of 15 items that describe different types of pain, divided into sensory and affective categories. Each item was rated from 0 to 3, where 0 represents “no pain”, 1 “mild pain”, 2 “moderate pain”, and 3 “strong pain”. The VAS scale consisted of a ten-centimeter-long horizontal line with two descriptors representing the extreme levels of pain (“absence of pain” and “agonizing pain”). A line was drawn on which the women marked their level of postoperative pain. We converted the measurements on the scale to the same number of points, ranging from 0 to 10. In the pain attribute scale, there are six attributes for pain characterization: 0 represents “absence of pain”, 1 “light pain”, 2 “unpleasant pain”, 3 “disturbing pain”, 4 “very strong pain”, and 5 “unbearable pain”.

### 2.4. Functional Recovery Assessment

Time to patient functional recovery was noted, including time to the first independent mobilization, time to the first oral intake, and lactation establishment. Additionally, we recorded the personal demographic (age, social status, education) and obstetric characteristics (parity and type of cesarean section) of every patient.

### 2.5. Statistical Analysis

Descriptive statistics were presented as mean values with standard deviation (SD) for numerical variables, or as absolute numbers with percentages for categorical variables. Differences between the GEA and RA (SA + EA groups) were presented as difference means with 95% confidence intervals and compared by Student’s t and chi-square test, for numerical and categorical data, respectively. Postoperative changes in pain characteristics were determined by repeated measurements ANOVA. The internal consistency of the SF–MPQ was assessed for multiple item scales by using Cronbach’s alpha coefficient (ranges from 0–1, the latter meaning perfect reliability). In all tests, the *p*-value < 0.05 was considered to be statistically significant. Statistical analysis was performed using IBM SPSS statistical software (SPSS for Windows, release 25.0, SPSS, Chicago, IL, USA).

## 3. Results

The study included 533 women that delivered by cesarean section. GEA was performed for 284 deliveries while RA was performed for 249 (SA for 162 and EA for 87). The mean age in the GEA group was 32.4 ± 4.5, while in the RA group, the mean age was 31.7 ± 4.8. Patients who underwent cesarean section under GEA were in gestational age of 38.4 ± 1.23 compared with 38.5 ± 1.35 in patients from the RA group. A total of 62 patients (21.8%) from the GEA group went to primary or secondary school, while the same number from the RA group was 46 (18.5%). Cesarean section was classified as urgent in 56 patients (19.7%) from the GEA group compared with 57 (22.9%) in the RA group. Age, gestational age, education level, and type of cesarean section did not differ between the groups. More details regarding study population has been described in detail elsewhere [15].

Postoperative pain characteristics in relation to applied GEA and RA are shown in Table 1. As measured by Cronbach’s alpha coefficient, the SF-MPQ questionnaire showed excellent reliability in all three measurements, i.e., 0.943, 0.905, and 0.915 after 2, 12, and 24 h, respectively. Cronbach’s alpha of the entire scale was 0.863, indicating scale reliability. Significant differences were found in the sensory and affective characteristics of postoperative pain between the two types of anesthesia within intervals of 2, 12, and 24 h after cesarean section (*p* < 0.001 for all respective intervals), with the GEA group having higher postoperative sensory and affective pain levels. Pain characteristics represented by the VAS scale showed significant differences regarding the type of anesthesia within all assessed intervals after cesarian section (*p* < 0.001 for all respective intervals), with the GEA group having higher postoperative VAS pain levels. A Pearson correlation coefficient higher than 0.8 showed a strong correlation between the SF-MPQ questionnaire with VAS for all three measurements. Both groups differed on the pain attribute scale intensity, with the GEA group having higher postoperative pain levels within intervals of 2, 12, and 24 h after cesarean section (*p* < 0.001). 

**Figure 1:**
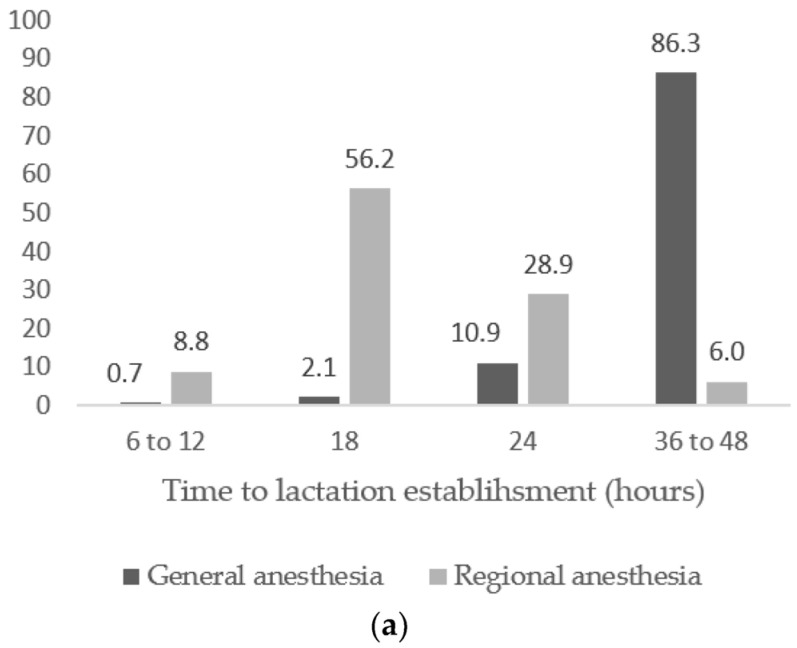

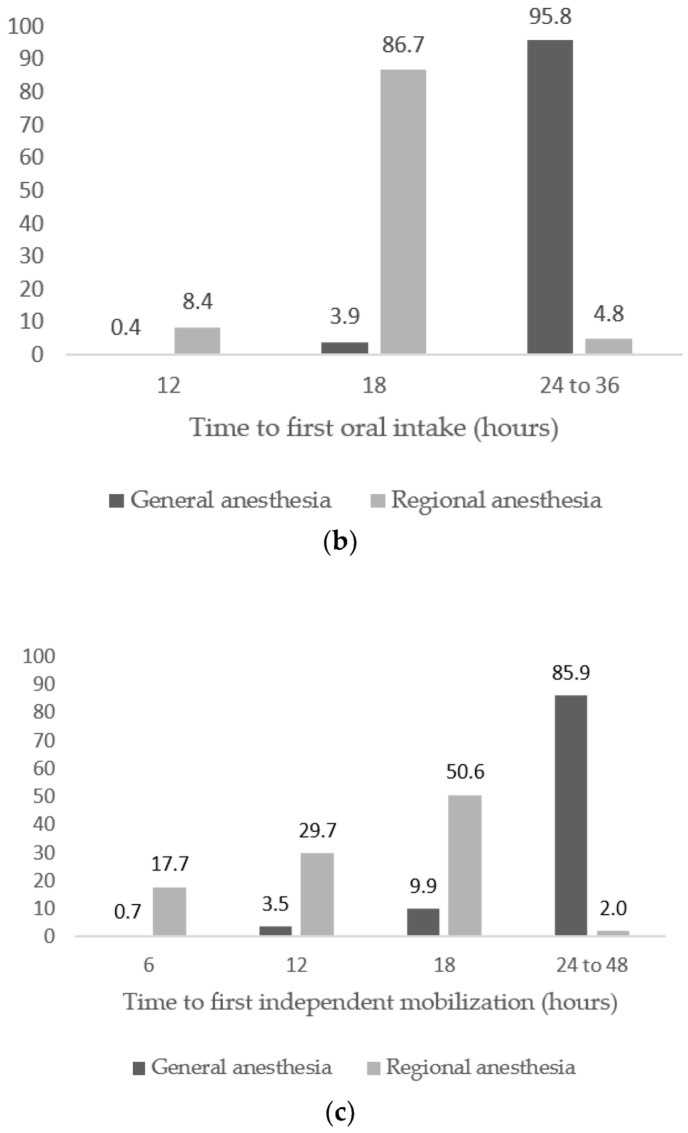
(**a**) Time to lactation establishment in hours according to anesthesia type; (**b**) time to first oral intake in hours according to anesthesia type; (**c**) time to first independent mobilization in hours according to anesthesia type.

In the GEA group, 86.3% of women established lactation 36 to 48 h after cesarean section, in contrast to the RA group, where 56.2% and 28.9% of women established lactation after 18 and 24 h, respectively. In the GEA group, 95.8% of women had their first peroral intake 24–36 h after birth, in contrast to the RA group, where 86.7% of women had peroral intake after 18 h. Additionally, the application of GEA resulted in 85.9% of women taking their first independent mobilization 24–48 h postoperatively, in contrast to the group receiving RA, where 29.7% of women established their first independent mobilization after 12 h, and 50.6% of them after 18 h (Figure 1).

## 4. Discussion

In this study, we aimed to compare the effects of RA and GEA on postoperative analgesic requirements and pain relief in women delivering by cesarean section. GEA had higher postoperative pain levels on SF–MPQ, VAS, and the pain attribute scale. Additionally, RA was associated with faster first independent mobilization and faster establishment of lactation.

Even though it is not considered a major procedure, the cesarean section is ranked ninth among 179 different procedures according to a recent study assessing postoperative pain intensity [2,17]. Anesthesia has an immense impact on the patient’s perception of postoperative pain, recovery time, and care for the newborn, and given that the cesarean section is one of the most common procedures, there has been increased interest in postoperative pain relief. The question of superiority stirs up controversy and remains undefined when comparing anesthesia in terms of post-cesarean analgesia. RA is the most popular type of anesthesia for the cesarean section because it is easy to perform and has the lowest percentage of complications. The most common complication is hypotension, which is more often present in SA compared with EA. It is preferable as it enables the mother to be present at the birth of her child and establish contact with the newborn from the first moments of life.

Kessous et al. [12] found that postoperative pain scores were comparable between GEA and spinal anesthesia. Interestingly, the pain scores in women who received GEA were significantly lower after 8 h postoperatively; however, this trend reversed in favor of spinal anesthesia after 48 h. An important finding was that postoperative analgesia requirements were higher in patients who received GEA [12]. These findings could be due to the relatively short effect of the anesthetic drugs used for spinal anesthesia (fentanyl) compared with drugs used for GEA. On the other hand, our study demonstrated significantly lower pain scores for RA across all assessed time intervals (2 h, 12 h, 24 h). These differences between studies might be related to the inclusion of epidural anesthesia in the RA group, as well as differences in multimodal approaches used for post-cesarean pain relief. Similar results were found in a Malaysian study where GEA and longer procedure durations were independent predictors of post-cesarean pain intensity [18].

Usage of GEA for cesarean sections has significantly declined in recent years [19,20,21]. This trend corresponds with dramatic decreases in anesthesia-related maternal mortality. [22]. As we previously mentioned, some risk factors determine the type of anesthesia for cesarean section. For example, severe heart valve stenosis, morbidly adherent placenta, or coagulation factor deficits are absolute indicators for the usage of GEA [23]. Because of its rapid and predictable effects, GEA is sometimes a preferable type of anesthesia for urgent cesarean sections [24]. More specifically, some urgent conditions (placental abruption or umbilical cord prolapse) can increase the rate of GEA in cesarean section to up to 20% [25]. Our study shows that urgent cesarean sections accounted for 19.7% of the GEA and 22.9% of the RA administered. The increasing trend in RA usage in an emergency cesarean section is confirmed by the results from numerous studies, such as ones from Italy and the United Kingdom [26,27]. The most frequently used technique was SA (94.1%), due to its simplicity, ease of administration, and faster onset of action. Nevertheless, The Society for Obstetric Anesthesia and Perinatology (SOAP) deems that the percentage of general anesthesia for cesarean section should be lower than 5% [28]. The Royal College of Anaesthetists considers the same percentage for the cesarean section classified as urgent [27].

To promote early recovery and optimize the parturient’s ability to care for her newborn baby, high-quality pain relief is of great importance after a cesarean section procedure. Postpartum pain is composed of not only nociceptive and neuropathic pain, but also four other components: sensory, affective, cognitive, and behavioral. We primarily examined the sensory and affective dimensions of pain at predetermined intervals of 2, 12, and 24 h postoperatively, using the VAS and SF-MPQ. Both sensory and affective characteristics differences were present between the GEA and RA across all time intervals. Furthermore, pain characteristics based on VAS scales within the same intervals showed significant differences. Almost three decades ago, Wang et al. [29] concluded a decrease in postoperative morphine requirement in patients who underwent cesarean section under spinal or epidural anesthesia compared with those under GEA. Not long after, Lertakyamanee [30] reported that women were satisfied with RA, and that RA was a better choice of anesthesia for cesarean section than GEA if adequate explanation and perioperative care are provided.

Even though different methods of analgesia are currently in use, pain relief and patient satisfaction are still inadequate in many cases [31,32,33]. Because of the fear of possible side-effects, pain after cesarean section often stays under-treated but also underestimated [34]. Therefore, continuous research about pain relief after cesarean delivery must be performed in order to prioritize maternal and newborn safety in the first place and to accelerate parturients’ recovery and ability to return to daily functional activities.

Another controversial topic with unclear results concerns breastfeeding after cesarean delivery. In the early postpartum period, the time of lactation establishment is vital for both mother and newborn. A recent Swedish study marked cesarean delivery as one of the main factors for exclusive breastfeeding lasting less than two months after delivery [35]. Moreover, anesthesia type during the procedure and its effect on breastfeeding are also imprecise. Nonetheless, the pain undoubtedly affects breastfeeding, and anesthesia directly affects sensory and affective pain characteristics. Babazade et al. [6] found significant breastfeeding deterioration, with an increase in post-cesarean pain. An increase in pain scores after the procedure was associated with a slight reduction in breastfeeding quality. One Turkish study revealed that women who delivered by cesarean section under GEA had the highest rate of breastfeeding problems compared with vaginal deliveries and cesarean sections under RA [36]. The opioids given during GEA affect a newborn’s neurobehavior, and also result in difficulty for mother when positioning for breastfeeding, providing a possible explanation of these results [36].

A recent meta-analysis showed that early oral feeding after cesarean section is not associated with the risk of postoperative complications, while also supporting a return to normal bowel function. In this meta-analysis, oral intake was provided within 6–8 h after the procedure [37]. The important finding of the our study is that in the GEA group, 95.8% of women had their first oral intake 24–36 h after birth, in contrast to the RA group, where 86.7% of women had peroral intake after 18 h. Additionally, results of our study showed that the application of GEA mostly resulted in mobilization establishment 24 to 48 h after birth, while RA resulted in mobilization establishment after 12 to 18 h. Preventing thrombophlebitis and numerous systemic complications, and improving the blood supply to various tissues, remain the main purposes of early verticalization [38]. Ghaffari et al. showed that women who chose spinal anesthesia modality more often reported having no pain, no problem with self-care, and mobility 24 h after cesarean section [39], thus favoring spinal anesthesia as the technique of choice for cesarean section. Our results further confirm the opinion that RA provides better pain management, mobility, and a faster return to activities for new mothers [39].

Our study has some limitations. Firstly, the results of this study represent a single-center experience. Since the University of Belgrade consists of two University Clinics for Obstetrics and Gynecology, further research should include the participants from these two centers. Moreover, research should include smaller Serbian hospitals for more representable and applicable results. We think that the inclusion of both University Clinics and local obstetric departments would significantly affect the percentage of GEA usage, since SA or EA is not available in many smaller hospitals with obstetrical departments. Another limitation is the lack of exact diagnosis as an indication for emergency cesarean section. Inclusion of these indications would allow us to re-evaluate the decisions for urgent procedures, thus providing a better insight into possible unnecessary usage of GEA.

To our knowledge, this is the first study of its kind in Serbia. Its results support the PROSPECT (systematic review utilizing procedure-specific postoperative pain management) recommendations for postoperative analgesia after cesarean section, which propose a multimodal pre-, intra- and postoperative analgesic strategy, while balancing the degree of pain after surgery and the invasiveness of the analgesic intervention [34]. Given the importance of pain management during and after cesarean section, we think this research will encourage multicenter, national studies, to provide nationwide accepted protocols for post-cesarean analgesia.

## 5. Conclusions

Despite major efforts, post-cesarean pain remains the greatest concern among women undergoing cesarean delivery, closely followed by the adequate and immediate care of the newborn. In this study, the application of RA presented superior postoperative pain relief, resulting in earlier mobilization, shorter time to first oral food intake, and faster lactation onset in contrast to GEA. Further research on this topic is essential to provide optimal and individual pain management after a cesarean section.

## Figures and Tables

**Table 1:** Pain characteristics after cesarean section according to anesthesia type.

Pain Characteristics	GEA (n = 284)	RA (n = 249)	Difference	95% CI for Difference	*p*
SF–MPQ Sensory					
2 h	23.22 ± 4.31	11.90 ± 4.13	11.32	10.60 to 12.04	<0.001
12 h	17.52 ± 3.77	7.67 ± 3.63	9.84	9.21 to 10.47	<0.001
24 h	13.42 ± 3.87	3.44 ± 2.24	9.97	9.43 to 10.52	<0.001
SF–MPQ Affective					
2 h	3.66 ± 2.17	0.24 ± 1.05	3.42	3.12 to 3.71	<0.001
12 h	0.76 ± 1.28	0.18 ± 0.76	0.58	0.40 to 0.76	<0.001
24 h	0.31 ± 0.82	0.08 ± 0.70	0.23	0.10 to 0.36	0.001
SF–MPQ Total					
2 h	26.88 ± 5.59	12.14 ± 4.43	14.74	13.87 to 15.61	<0.001
12 h	18.27 ± 4.25	7.85 ± 3.78	10.42	9.73 to 11.11	<0.001
24 h	13.73 ± 4.16	3.53 ± 2.57	10.20	9.60 to 10.80	<0.001
VAS					
2 h	8.77 ± 0.91	6.06 ± 1.12	2.71	2.53 to 2.88	<0.001
12 h	6.74 ± 0.87	4.00 ± 1.14	2.74	2.57 to 2.91	<0.001
24 h	5.19 ± 0.91	2.28 ± 1.25	2.92	2.73 to 3.10	<0.001
Pain attribute scale					
2 h	3.94 ± 0.55	2.51 ± 0.60	1.43	1.33 to 1.53	<0.001
12 h	2.92 ± 0.45	1.77 ± 0.52	1.15	1.07 to 1.23	<0.001
24 h	2.01 ± 0.28	1.09 ± 0.57	0.92	0.85 to 1.00	<0.001

## Data Availability

The data that support the findings of this study are available on request from the corresponding author.
